# Screening of Natural Products Targeting SARS-CoV-2–ACE2 Receptor Interface – A MixMD Based HTVS Pipeline

**DOI:** 10.3389/fchem.2020.589769

**Published:** 2020-11-19

**Authors:** Krishnasamy Gopinath, Elmeri M. Jokinen, Sami T. Kurkinen, Olli T. Pentikäinen

**Affiliations:** Faculty of Medicine, Integrative Physiology and Pharmacology, Institute of Biomedicine, University of Turku, Turku, Finland

**Keywords:** COVID-19, mixed solvent molecular dynamics simulation, natural product, spike protein, ACE2

## Abstract

The COVID-19 pandemic, caused by novel severe acute respiratory syndrome coronavirus 2 (SARS-CoV-2), is a severe global health crisis now. SARS-CoV-2 utilizes its Spike protein receptor-binding domain (S-protein) to invade human cell through binding to Angiotensin-Converting Enzyme 2 receptor (ACE2). S-protein is the key target for many therapeutics and vaccines. Potential S-protein–ACE2 fusion inhibitor is expected to block the virus entry into the host cell. In many countries, traditional practices, based on natural products (NPs) have been in use to slow down COVID-19 infection. In this study, a protocol was applied that combines mixed solvent molecular dynamics simulations (MixMD) with high-throughput virtual screening (HTVS) to search NPs to block SARS-CoV-2 entry into the human cell. MixMD simulations were employed to discover the most promising stable binding conformations of drug-like probes in the S-protein–ACE2 interface. Detected stable sites were used for HTVs of 612093 NPs to identify molecules that could interfere with the S-protein–ACE2 interaction. In total, 19 NPs were selected with rescoring model. These top-ranked NP–S-protein complexes were subjected to classical MD simulations for 300 ns (3 replicates of 100 ns) to estimate the stability and affinity of binding. Three compounds, ZINC000002128789, ZINC000002159944 and SN00059335, showed better stability in all MD runs, of which ZINC000002128789 was predicted to have the highest binding affinity, suggesting that it could be effective modulator in RBD-ACE2 interface to prevent SARS-CoV-2 infection. Our results support that NPs may provide tools to fight COVID-19.

## Introduction

The severe acute respiratory syndrome Coronavirus-2 (SARS-CoV-2), has led to a global pandemic, as it had spread rapidly around the world. The entire scientific community is in an urge to find a therapeutic solution to reduce the spread and severity of COVID-19 infections. In many countries, traditional medicines have been in use to fight against COVID-19. The traditional, complementary and alternative medicines have many benefits (World Health Organization, 2020). The traditional Chinese medicine (TCM) can effectively contribute to COVID-19 treatment as an alternative measure. In China, The TCM has been in use along with the conventional Western antiviral medicine for the treatment of COVID-19, many clinical trials are in progress to test the efficacy and safety in COVID-19 treatment (Lim, 2020; Ling, 2020). Yang et al. (2020) reported that 85% of SARS-CoV-2 infected patients in China are receiving TCM treatment (Yang et al., 2020). A clinical trial is registered in India to study the effect of nutraceutical formulations to fight against SARS-CoV-2. Another clinical trial is proposed to study the effect of natural product-based oral spray with curcumin and artemisinin for the treatment of COVID-19. Food supplements also contribute to a therapeutic solution for COVID-19. Researchers from Australia, Egypt and Saudi Arabia proposed to clinically test the Zinc, Natural Honey and oral nutrition supplements respectively to fight against SARS-CoV-2. Besides that, in the US and Spain, a clinical trial studies the effect of micronutrients and even resistant potato starch in clinical recovery (Koe, 2020a,b).

SARS- CoV-2 has four structural and seven non-structural proteins (Shereen et al., 2020). S-protein is one of the structural proteins and is present on the surface of the coronavirus. S-proteins are highly glycosylated. These S-proteins are the protrusions on the virus, and the spike is clove shaped with three receptor-binding S1 heads attaching to the top of a trimeric membrane fusion S2 stalk, these protrusions on the surface resemble a crown. The S-protein plays a vital role in invading the host cells (Walls et al., 2020). Previously, it has been reported that angiotensin-converting enzyme 2 (ACE2) is essential for SARS-CoV-2 infection, acting as its effective host receptor (Kuba et al., 2005). Both SARS-CoV and SARS-CoV-2 share similar cell entry mechanism by binding with ACE2 located on the surface of the host cell. The ACE2 receptor is widely expressed in lungs, guts, kidney, cardiovascular system, central nervous system and adipose tissue (Mahmoud et al., 2020). The S1 subunit contains a receptor-binding domain (RBD) that recognizes ACE2 (Lin et al., 2020). The RBD of S1 subunit undergoes conformational changes upon recognizing ACE2. The RBD stands up and keeps the S1 domain in an open conformational state to initiate the attachment of the virus to the human ACE2. This conformational change of RBD results in the fusion with the host cell membrane through the S2 subunit of the Spike protein. The S2 subunit helps the virus in conformational changes during the fusion process of the virus after endocytosis, by then the pH levels on the surface of the host cell gets reduced and helps in the intervention of virus. (Laha et al., 2020; Shang et al., 2020). This functional mechanism of RBD of the S-protein provides the framework for the design of the inhibitors to prevent the entry of the virus into a host cell, and thus, curbs further infections in the host.

The S-protein is considered as the potential target for therapeutic intervention and vaccination. The main interest of this study was to search for NPs that could inhibit the SARS-CoV-2 interaction with human cells, and thus, prevent the replication of the virus. NP-databases have numerous bioactive compounds with known antiviral properties. NPs have been practically always used for the treatment of various infections (Gopinath et al., 2020). In the context of SARS-CoV-2, NPs have contributed effectively in the past to treat severe acute respiratory syndrome caused by SARS-CoV and MERS-CoV. Accordingly, in the present alarming situation, NPs would be an obvious resource to identify treatments against SARS-CoV-2 (Antonio et al., 2020). For example, NPs have the potential to block the recognition site of the HSPA5 cell-surface and compete for the viral spike recognition (Antonio et al., 2020; Elfiky, 2020), two natural products, thioflexibilolide A and candidine, potentially interact with RBD domain of S-protein (Chen et al., 2020), and herb-derived naturally occurring compounds sinigrin, indigo, aloe-emodin, hesperetin, quercetin, epigallocatechin gallate, herbacetin, rhoifolin and pectolinarin have potential to inhibit the SARS 3CLpro activity in SARS-CoV-2 (Chen et al., 2020; Elfiky, 2020; Yang et al., 2020). Also, NPs have been reported to have effect potential in the treatment of SARS-CoV-2 (Ling, 2020; Steele, 2020). Computer-aided drug screening has been performed to find effective molecules for fighting against SARS-CoV-2 that is causing current pandemic (Chen et al., 2020).

Supercomputers have been in use across the world to accelerate the COVID-19 drug search. Recent advancement in computational facilities has increased the efficient usage of various MD simulation techniques in drug search (Amaro et al., 2008, 2018; Salmaso and Moro, 2018). Mixed solvent molecular dynamics simulations (MixMD) is a cosolvent simulation technique for identification of binding hotspots such as orthosteric, allosteric and cofactor binding sites as well as protein-protein interaction (PPI) sites (Ghanakota and Carlson, 2016; Ung et al., 2016). In addition, the conformational changes of these sites can be observed to find suitable protein conformations for docking. Our natural product search pipeline combines MixMD with high-throughput virtual screening (HTVS) in the initial screening. Here, we used MixMD to detect potential inhibitor binding sites on RBD—ACE2 protein-protein interaction (PPI) interface. The identified binding site was used for the HTVS with various natural product databases. Furthermore, rescoring of docking results jointly with MD simulations, and binding energy calculations were carried out to identify potent NPs that could block the S-protein—ACE2 interaction.

## Methods

### MixMD Simulations

In MixMD simulations, drug-like organic probe molecules are added to the solvent, and their localization during simulations is observed to detect possible small molecule binding sites on the protein surface (Ghanakota and Carlson, 2016; Ung et al., 2016). Crystal structure of SARS-CoV-2 S-protein complexed with ACE2 (PDB: 6M0J) was obtained from Protein Data Bank in 2.45 Å resolution (Lan et al., 2020). ACE2, ions and crystal waters were deleted. Three different probes were used in MixMD simulations: pyrimidine (1P3), acetonitrile (ACN) and isopropanol (IPA). RBD was solvated in 5% v/v ratio of the probe to water with each probe. Tleap in AMBER18 (Case et al., 2020) was used for simulation setup. Hydrogens were added, and disulfide bonds and histidine protonation were assigned. A layer of probe molecules was added after which the system was solvated with enough TIP3P (Jorgensen et al., 1983) water to obtain the correct probe-water ratio. Protein was parameterized with ff14SB force field (Maier et al., 2015). Parameters from GLYCAM_06j-1 force field were used for N-acetyl-D-glucosamine and glycosylated asparagine (Kirschner et al., 2008). Parameters validated for MixMD simulations in TIP3P water were used for 1P3, ACN and IPA (Lexa et al., 2014).

CUDA implementation of PMEMD was used in all simulations (Götz et al., 2012; Salomon-Ferrer et al., 2013). NPT simulations were run using 2 fs timestep. Temperature and pressure were maintained at 300 K and 1 atm, respectively, using Andersen thermostat (Andersen, 1980). SHAKE algorithm was used to restrain bonds to hydrogen atoms (Ryckaert et al., 1976). Short-range electrostatics cutoff was set to 10 Å, and Particle Mesh Ewald was used for long-range electrostatics (Darden et al., 1993; Essmann et al., 1995). Energy minimization and gradual heating were conducted for all systems. Protein backbone was restrained during equilibration runs. After gradually decreasing the backbone restraints, 1.4 ns equilibrations were run without restraints. Production simulations were continued until 100 ns and repeated ten times per probe, resulting in 3 μs of total simulation time.

### Probe Pose Clustering

Last 25 ns of each simulation run was included in probe pose clustering. Probe occupancy maps were generated to identify sites on the RBD surface where probe molecules have high residence times. Grid function in cpptraj (Roe and Cheatham, 2013) was used with 0.5 Å spacing to generate the occupancy maps. The maps of different probes were visualized and combined in Pymol 2.3.0 (The PyMOL Molecular Graphics System, Version 2.0 Schrödinger, LLC). σ values of probe densities were adjusted as described by Ghanakota and Carlson (2016). Higher σ value enables visualization of spots with the highest probe residence times while the sites where probes are rapidly exchanged with water get disposed. Based on the occupancy map visualization, the residues that are close to or in contact with the probe densities were identified and used in probe pose clustering.

The most common probe poses at the high occupancy sites were obtained by root-mean-square deviation (RMSD)-based clustering of probes using cpptraj. Probe molecules within <3 Å of residues around the observed probe densities were extracted from all simulation snapshots. Each probe molecule was written in its PDB file, and all the probes were given the same residue and atom numbers to enable clustering in cpptraj. The probe files were loaded to cpptraj, and clustering was performed with the average-linkage method using epsilon value 4 Å. After this, RBD structures related to the obtained centroid probes were searched from the simulation trajectories to evaluate their use in molecular docking.

### Ligand Preparation

The natural product database used in HTVS consists compounds from ZINC Biogenic (http://zinc.docking.org/substances/subsets/biogenic), FooDB Version 1.0 (https://foodb.ca), Molport Natural Compound and Natural-Like Compound Database (www.molport.com) and Super Natural II database (Banerjee et al., 2015). All compounds were downloaded as 2D structural data and were converted to 3D format with OPLS3 charges and tautomeric states at pH 7.4 using LIGPREP in MAESTRO 2020-1 (Schrödinger, LLC, New York, NY, United States, 2020). Molecules that possess more than ten rotatable bonds or molecular weight that exceeds the range of 150–550 g/mol were excluded from the dataset with LIGFILTER in MAESTRO. Molport, ZINC and Super Natural II databases were also filtered from the molecules exceeding partition coefficient (logP) of 5.7 calculated with QIKPROP in MAESTRO. Conversion of molecules to SYBYL MOL2 format was done with MOL2CONVERT in MAESTRO.

### Virtual Screening and Rescoring

Protein conformation from probe pose clustering of MixMD was used for HTVS. HTVS of NPs was performed with PLANTS software (Korb et al., 2009). The docking site was defined as a sphere with 12 Å radius from Arg403 of RBD, Chemplp scoring function and search speed “speed1” were used in screening. Five best-scored conformations of each compound were kept with cluster RMSD 3.0.

Docking results were rescored with the negative image-based (NIB) rescoring method using the programs PANTHER (Niinivehmas et al., 2015) (version 0.18.19) and ShaEP (Vainio et al., 2009) (version 1.3.1). PANTHER was used to generate a NIB-model that describes the shape and electrostatic properties of an optimal ligand for the binding pocket. Extremely fast molecular similarity screening was then performed with ShaEP with the option “noOptimization” to score the ligand poses generated by the docking program. PANTHER rescoring has resulted in significant enrichment of active compounds in VS with multiple protein targets (Kurkinen et al., 2018, 2019). Furthermore, 1P3 molecules obtained by probe pose clustering were incorporated to the NIB-model to favor compounds forming similar interactions in the high occupancy areas. This fragment-based approach for NIB-screening has been previously utilized in the discovery of active inhibitor molecules (Jokinen et al., 2019). In NIB-model generation, suitable coordinate points were used to obtain small molecule like entities into the identified cavity. Face-centered cubic packing method was used. In models where 1P3 molecules were included, their partial charges were assigned similarly as for the rest of the model. Those nitrogen atoms of 1P3 that were buried were replaced with neutral carbon atoms.

Top ranking compounds from rescoring were inspected in Maestro (Schrödinger Release 2020-1: Maestro, Schrödinger, LLC, New York, NY, 2020). Top ranking natural compounds with satisfying ESP and Shape similarity score from rescoring calculation were selected for further computational validation.

### Molecular Dynamics (MD) Simulation and Post-MD Analysis

Classical MD simulations were performed with AMBER18 to ensure the binding stability of 19 top-ranked compounds from rescoring (Table 1). Protein-ligand complexes were solvated in a cubic box of TIP3P water extending 10 Å from protein atoms in each dimension. Otherwise, the same simulation protocol and settings were used as in the MixMD simulations. General AMBER force field was used to obtain parameters for the ligands (Wang et al., 2004). Ligand topology files and atomic charges were generated with antechamber, using AM1-BCC charge method (Wang et al., 2006). For each protein-ligand complex, three 100 ns repeats were performed, and post-MD analysis was performed with the snapshots of the trajectory ensembles. Principal component analysis (PCA) was performed using CPPTRAJ on three replicates of MD simulation of each complex (Galindo-Murillo et al., 2014). The coordinate covariance matrix of all-atom was calculated for the raw trajectory of all three simulations for each complex, and first three eigenvectors were obtained using the matrix, and principal component data was visualized with the Normal Mode Wizard (NMWiz) plugin of VMD. Variation in the ligand location and its motion was examined using the porcupine plot of the first three eigenvectors of the entire simulation of each complex. Further, the stability of compound binding was also checked by hydrogen bond analysis, and binding affinity calculation were performed using MMPBSA.py (Miller et al., 2012).

**Table 1:** The selected compounds from HTVS and their PANTHER/ShaEP-based rescoring.

**Database ID**	**ESP similarity score**	**Shape similarity score**
ZINC000002155511	0.210	0.697
MolPort-002-515-240	0.214	0.692
SN00059335	0.203	0.682
ZINC000002151580	0.203	0.675
ZINC000072325799	0.205	0.666
SN00236224	0.209	0.661
ZINC000002159944	0.219	0.649
MolPort-021-745-932	0.203	0.648
ZINC000002108239	0.222	0.647
ZINC000096296967	0.206	0.638
MolPort-027-852-900	0.203	0.637
ZINC000002108298	0.212	0.635
ZINC000095559555	0.203	0.629
ZINC000002102314	0.209	0.628
SN00341524	0.210	0.621
FDB023015	0.300	0.619
MolPort-027-852-870	0.209	0.618
ZINC000002114285	0.204	0.616
ZINC000002128789	0.217	0.615

*Compound ID prefix depicts its source (ZINC0000-ZINC biogenic; FDB- FooDB; SN- Super Natural II database; MolPort- Molport database)*.

### Protein-Protein Interaction Analysis

Protein-protein Interaction analysis was performed to examine the effect of the selected compound in the ACE2–Spike protein interface region. Ligand bound RBD structure was obtained from the final frame of three replicates. Ligand bound structures were superimposed with the crystal structure of ACE2–RBD using atom-pairs method and ACE2–ligand-bound RBD complex was obtained by replacing the RBD with ligand-bound RBD. The structures were further prepared, and atom clashes were removed using the protein preparation wizard. The entire preparation was done in Maestro and Optimized Potentials for Liquid Simulations (OPLS)-2005 force field was used for minimization (Schrödinger Release 2020-3: Maestro, Schrödinger, LLC, New York, NY, 2020). Molecular dynamics simulations were performed for ACE2 with RBD domain, and ACE2 with ligand-bound RBD. Each complex was minimized and solvated with TIP3P water model in an orthorhombic box with a distance of 10 Å. The whole system was neutralized by adding Na^+^ ions. The system was prepared using OPLS-2005 force field, and subjected to MD simulation using Desmond (Desmond Molecular Dynamics System, D. E. Shaw Research, New York, NY, 2020 Maestro-Desmond Interoperability Tools, Schrödinger, New York, NY, 2020) under the NPT ensemble with the temperature of 300 K and pressure of 1.01325 bar followed by relaxation (Ponder and Case, 2003; Bowers et al., 2006). 100 ns MD simulations were carried out for each protein-ligand bound complex. Finally, the snapshots of the complex from the trajectory were extracted using “trj2mae.py” script. Protein-Protein interaction fingerprint analysis was performed with BioLuminate by using a distance cutoff of 4 Å (Schrödinger Release 2020-3: BioLuminate, Schrödinger, LLC, New York, NY, 2020).

## Results and Discussion

### Detection of the Binding Site in the S-Protein—ACE2 Interface

**Figure 1:**
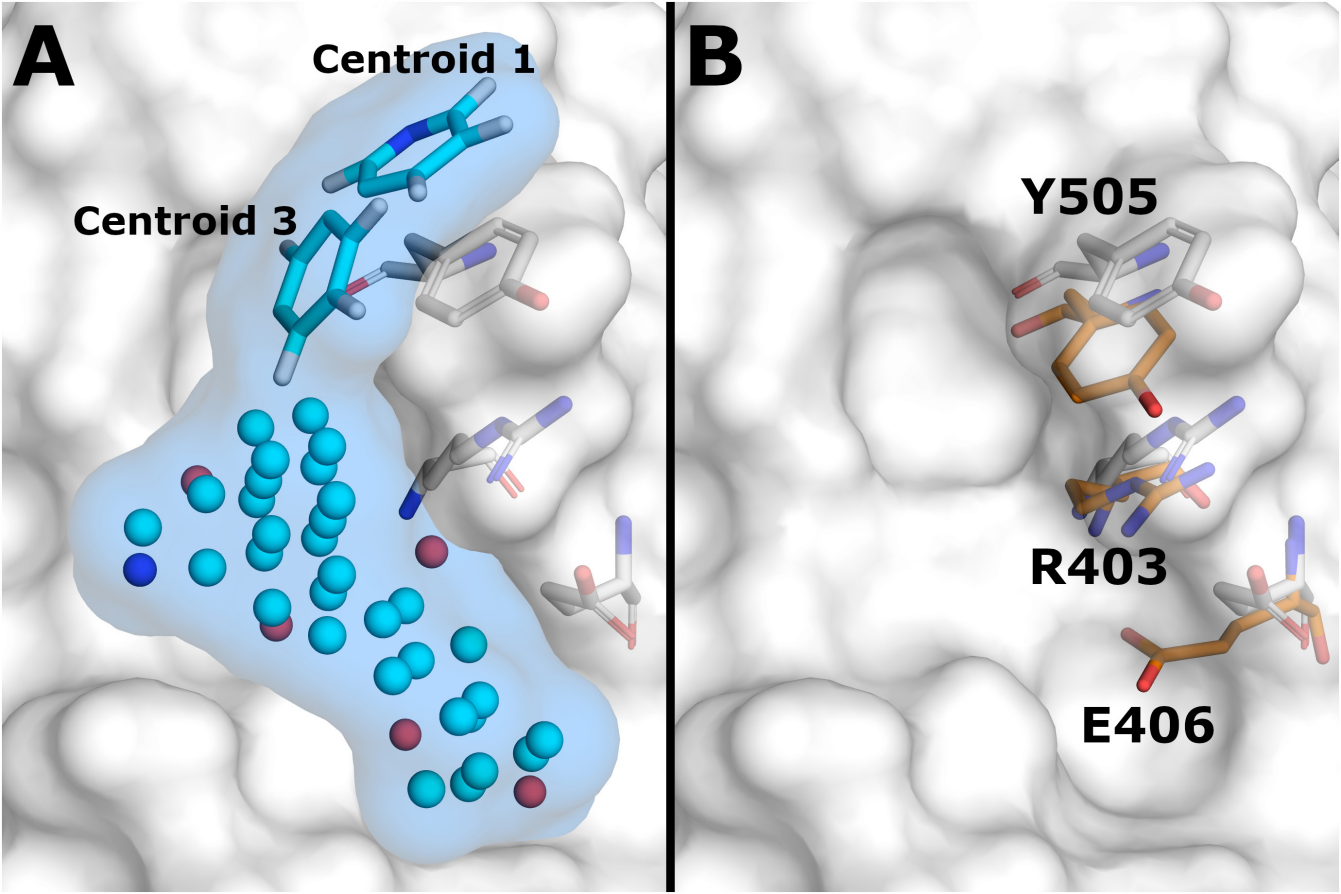
Docking conformation of ACE2 binding interface of RBD. **(A)** NIB-model (light blue surface) used in rescoring of docking results. Model atoms are shown as cyan (neutral), blue (positively charged), and red (negatively charged) spheres. Centroid probes included in the model are shown as sticks (C = cyan, N = blue, H = white). **(B)** Comparison of three ACE2 interface amino acid conformations between crystal structure (sticks, orange C atoms) and energy minimized MD simulation structure (sticks, white C atoms) of RBD. Simulation structure of RBD related to the probe centroid 3 is shown as white surface representation in both figures.

To identify the suitable druggable site with binding hot spots from the S-protein—ACE2 interface, MixMD simulations with different probes were used. The identified stable probe poses were used as seeds in the HTVS. Probe pose clustering analysis was focused on 1P3 as it showed the highest occupation at the ACE2 binding interface of RBD (Jokinen et al., submitted). Two 1P3 poses were identified that overlapped with the region known to be occupied by Lys353 of ACE2 (centroids 1 and 3), suggesting a possible mechanism for small molecule inhibition of ACE2 binding. Centroid 1 was the second and centroid 3 the fourth most common 1P3 pose at the ACE2 interface, and both were used in the HTVS rescoring model (Figure 1A). Both centroid probes showed a favorable binding mode by forming π-stacking interaction with Tyr505 in the area predicted to be crucial for inhibition. RBD structure related to centroid 3 was chosen for molecular docking as it had a groove-like shape at the PPI interface that could accommodate binding small molecules. Comparison with the crystal structure (PDB: 6M0J) showed that the residues Tyr505, Arg403, and Glu406 adopted conformations that increased the depth of this groove and expanded the space available for a possible small molecule inhibitor (Figure 1B).

### Screening of Potential Natural Products

Here, the goal was to identify NPs that would bind into S-protein and interfere with SARS-CoV-2 attachment to the host cell. The screened NP-library library consisted of ZINC biogenic (206,800 compounds) FooDB (18,477 compounds), Molport Natural Compound and Natural-Like Compound Database (119,054 compounds), and Super Natural II database (267,762). HTVS-docking was performed with this NP-database against the detected binding site in S-protein. Docking results were filtered by using rescoring with PANTHER/ShaEP-based NIB rescoring where both the shape and electrostatic potential (ESP) were compared between cavity-based NIB model and the docked molecule (Vainio et al., 2009; Niinivehmas et al., 2015; Kurkinen et al., 2018, 2019). Identified stable 1P3 poses were used in the NIB-models as they show the area where drug-like compounds could bind.

Interaction analysis was performed for the filtered compounds having ESP similarity score > 0.2 and Shape similarity score > 0.6, in NIB-model based rescoring, by using Ligand Interaction Diagram Panel in Maestro (Schrödinger Release 2020-2: Maestro, Schrödinger, LLC, New York, NY, 2020). Further, compounds interacting with residues in the interface region and that occupy the cavity region next to Tyr505 and Arg403 were selected (Table 1). Recent studies imply that the following residues Arg403, Asp405, Lys417, Asn439, Val445, Gly446, Tyr449, Tyr453, Lys455, Phe456, Tyr473, Ala475, Gly476, Glu484, Asn487, Tyr489, Gln493, Gln498, Gln493, Gln498, Thr500, Asn501, Gly502, Val503, Tyr505 mediates the fusion of SARS-CoV-2 with the cellular membrane through RBD-ACE2 interface (Brielle et al., 2020; Lan et al., 2020; Sun et al., 2020). Herein, selected NPs interacts with the surrounding amino acids by hydrogen bonds, π-stacking interactions, and salt bridges (Table 2). Further computational validation was extended to check the compound's affinity and stability from classical MD simulation and Principal component analysis and hydrogen bond analysis.

**Table 2:** Amino acid contacts of natural compounds in the S-protein–ACE2 interface region.

**Database ID**	**Hydrogen bond**	**Pi-Pi**	**Pi-Cation**	**Salt bridge**
ZINC000002155511	Arg403 (1), Gly496 (1), Asn501 (1)	Tyr505 (1)	Arg403 (2)	
MolPort-002-515-240	Gly496 (1)	Tyr505 (1)	Arg403 (1)	
SN00059335		Tyr505 (2)	Arg403 (2)	Arg403 (1)
ZINC000002151580	Gly496 (1), Asn501 (1)			
ZINC000072325799	Gln409 (1), Lys417 (1), Tyr453 (1), Asn501 (1)	Tyr505 (1)	Arg403 (1)	
SN00236224	Arg403 (1), Gln409 (1), Gly496 (1)			
ZINC000002159944	Arg403 (1), Gly496 (1), Tyr453 (1), Asn501 (1)	Tyr505 (2)	Arg403 (1)	
MolPort-021-745-932	Arg403 (1), Gln409 (1), Lys417 (1), Tyr453 (1)	Tyr505 (1)	Arg403 (1)	
ZINC000002108239	Arg403 (1)	Tyr505 (1)	Arg403 (1)	Arg403 (1)
ZINC000096296967	Arg403 (1), Gly496 (1)			
MolPort-027-852-900	Arg403 (1), Gly496 (1)			
ZINC000002108298	Arg403 (1)	Tyr505 (1)		Arg403 (1)
ZINC000095559555	Gly496 (1)			
ZINC000002102314	Arg403 (1), Tyr453 (1), Asn501 (1)	Tyr505 (3)	Arg403 (2)	
SN00341524	Gln409 (1), Lys417 (1), Gly496 (1), Tyr505 (1)	Tyr505 (1)		
FDB023015	Arg403 (1), Glu409 (1)			
MolPort-027-852-870	Gly496 (1), Asn 501 (1)	Tyr505 (1)	Arg403 (1)	
ZINC000002114285	Arg403 (1), Tyr453 (1), Asn501 (1)	Tyr505 (2)	Arg403 (2)	
ZINC000002128789	Arg403 (1)	Tyr505 (2)	Arg403 (2)	

*Values in the parenthesis represent the number of contacts with each residue*.

### Affinity and Stability of Selected Natural Products

**Figure 2:**
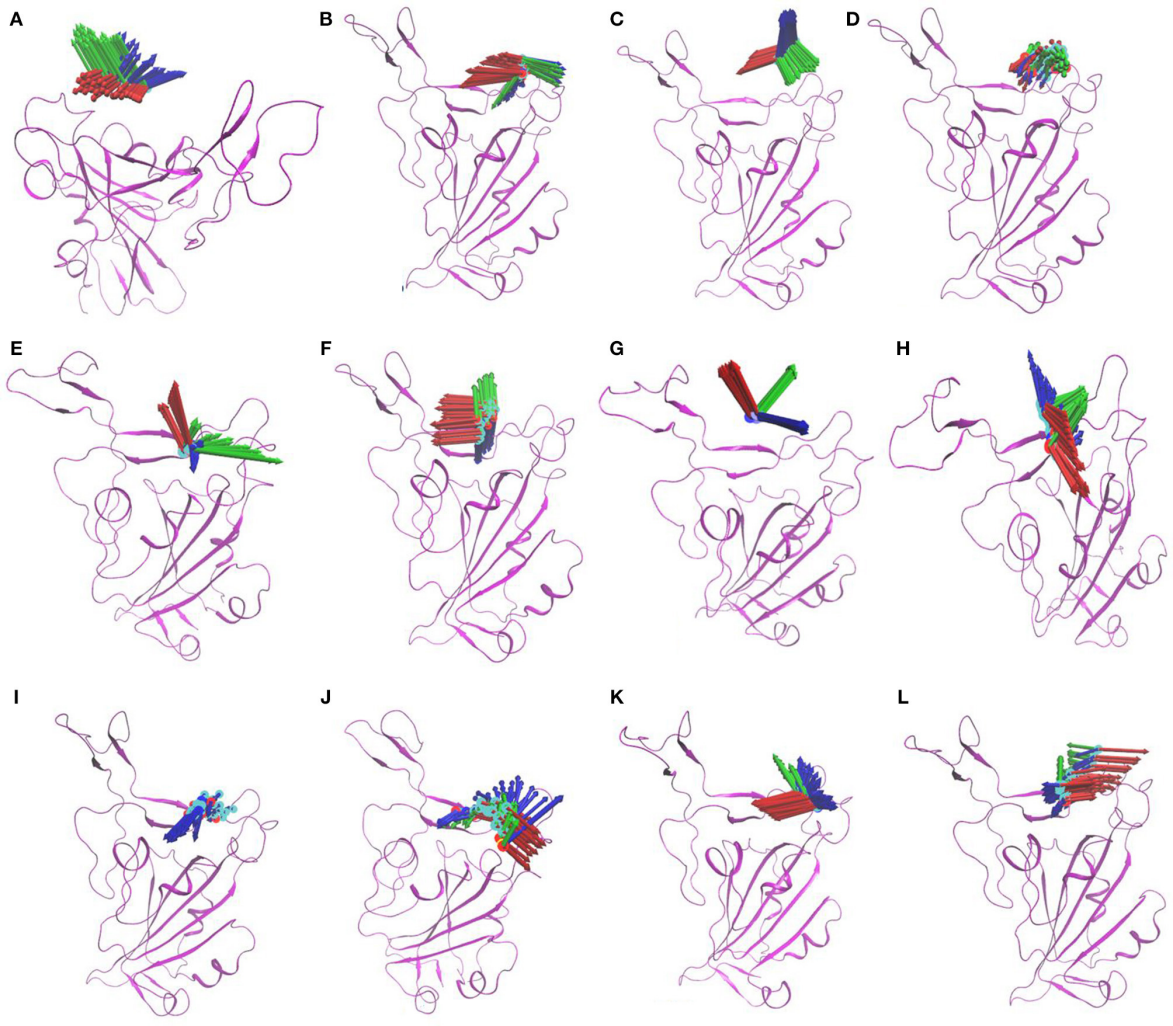
Porcupine plots of first three eigenvectors for three MD simulation replicates of selected compounds ZINC000002155511 **(A)**; SN00059335 **(B)**; ZINC000002151580 **(C)**; ZINC000002159944 **(D)**; MolPort-021-745-932 **(E)**; ZINC000002108239 **(F)**; ZINC000096296967 **(G)**; ZINC000002108298 **(H)**; ZINC000002128789 **(I)**; FDB023015 **(J)**; MolPort-027-852-870 **(K)**; ZINC000002114285 **(L)**. The arrows present on the protein complex indicate the direction and magnitude of the motion.

MD simulations were performed for selected NP—protein complexes for 300 ns (3 replicates of 100 ns) using Amber18, and trajectories of all replicates were used for PCA analysis. PCA systematically reduces the dimensionality of a complex system, and can characterize the cumulative and overall motion of the protein-ligand system (Bhutani et al., 2015). PCA is used to check the dominant modes of motion in a trajectory and variance in the data (Haider et al., 2008). First three PCs for each complex was obtained by diagonalizing the coordinate covariance matrix. Further, the analysis was focused on ensuring the stability using obtained PCs. A porcupine plot was drawn with first three eigenvector of each complex, and differences in the ligand location on generated averaged coordinates and motion was examined in VMD. The arrows in the porcupine plot represent the ligand direction and magnitude of the motion in three PC modes. Among all compounds, ZINC000002159944 and ZINC000002128789 had clearly lower magnitude of motion, and they remained located on the binding site in the generated averaged coordinates. The compounds SN00059335, ZINC000002108239, and FDB023015 were found to be in the binding site of the protein with less motion. Another five compounds, ZINC000002151580, MolPort-021-745-932, ZINC000002108298, MolPort-027-852-870, and ZINC000002114285, were located in the binding pocket with higher magnitude of the motion in all modes. Compounds ZINC000096296967 and ZINC000002155511 (Figures 2A–L) are slightly off from the binding pocket in the generated averaged coordinates, and they show a higher magnitude of the motion in all modes. In contrast, ZINC000096296967, SN00341524, ZINC000095559555, MolPort-002-515-240, ZINC000072325799, MolPort-027-852-900, and SN00236224 show higher deviation, and they moved out of binding site in the generated averaged coordinates. Thus, these seven compounds do not likely form strong enough interactions with S-protein to remain bound (Supplementary Figure 1).

**Figure 3:**
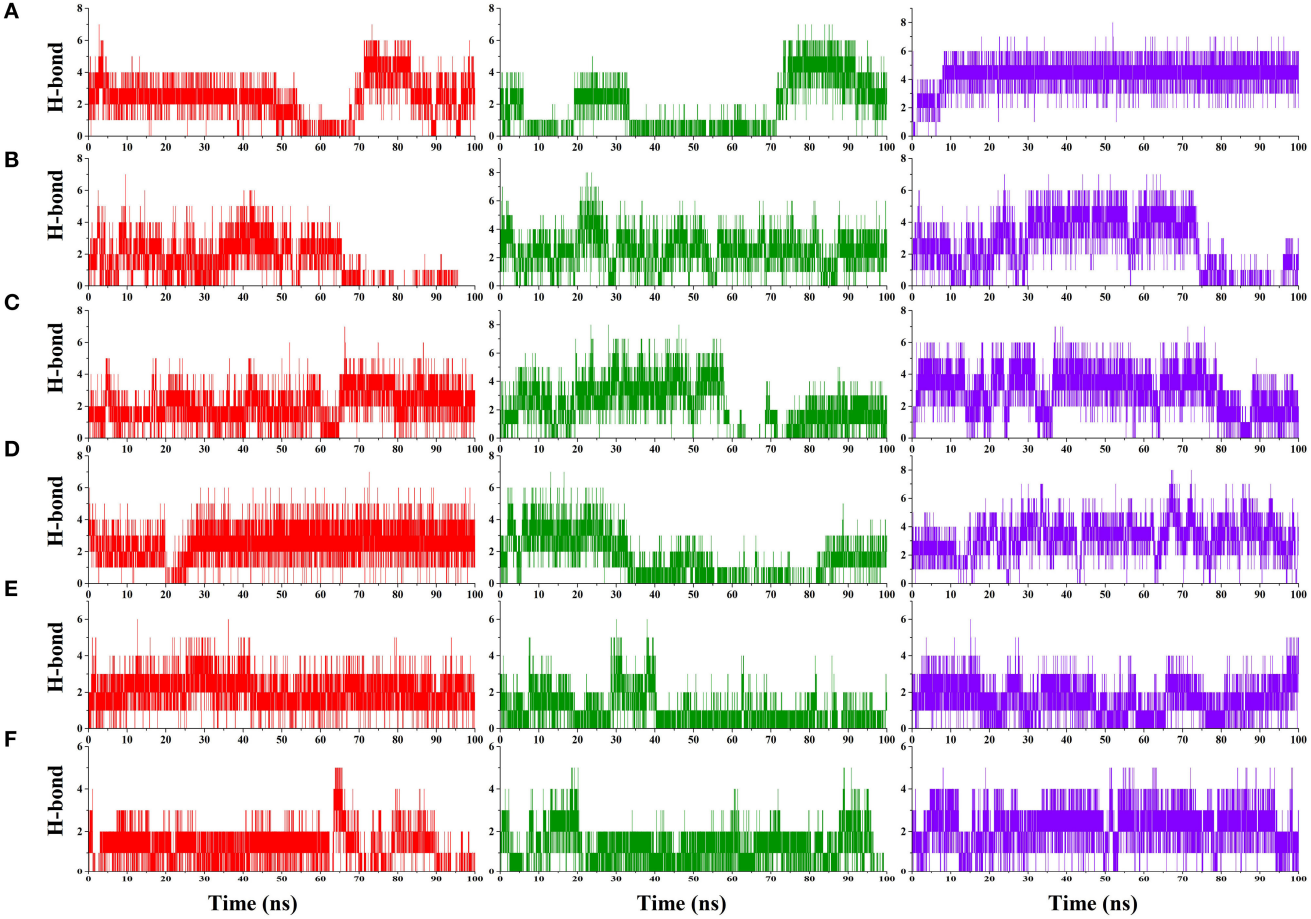
Hydrogen bond plot. Number of hydrogen bonds formed between S-protein and ZINC000002128789 **(A)**, ZINC000002114285 **(B)**, FDB023015 **(C)**, MolPort-021-745-932 **(D)**, ZINC000002159944 **(E)**, and SN00059335 **(F)** during entire simulation period, where compounds X axis shows Time in ns and Y axis shows the number of hydrogen bond formed between receptor and ligand in MD run 1 (Red) 2 (Green) and 3 (Blue).

Hydrogen bond analysis was carried to understand the stability of the compound binding to RBD. Time-dependent behavior of the hydrogen bonds was monitored, and the number of hydrogen bonds per frame was plotted. Compounds ZINC000002128789, ZINC000002114285, FDB023015, MolPort-021-745-932, ZINC000002159944, and SN00059335 (Figures 3A–F) showed sustained hydrogen bonds with RBD domain, and they all had more than 1 average hydrogen bond per frame in all three MD runs (Table 3). The porcupine plot also shows that these compounds were in the binding interface of the RBD domain. Remaining compounds, MolPort-002-515-240, MolPort-027-852-870, MolPort-027-852-900, SN00236224, SN00341524, ZINC000002102314, ZINC000002108239, ZINC000002108298, ZINC000002151580, ZINC000002155511, ZINC000072325799, ZINC000095559555, and ZINC000096296967 (Supplementary Figures 2, 3) do not show sustained hydrogen bond connections, and the average number of hydrogen bonds per frame is more than 1 in only one or two MD runs. Hydrogen bond-lifetime analysis was focused for top six stably binding compounds. This analysis depicts that during the simulations ZINC000002128789, ZINC000002159944 and SN00059335 form hydrogen bonds with ACE2-Spike protein binding interface residues, whereas, ZINC000002114285, FDB023015, and MolPort-021-745-932 also form hydrogen bonds with residues that are located outside of the binding site (Table 4).

**Table 3:** Calculated average hydrogen bond per frame for natural compounds in three MD runs.

**Compounds**	**MD run 1**	**MD run 2**	**MD run 3**
ZINC000002128789	2.39 ± 1.32	1.74 ± 1.70	4.36 ± 1.10
ZINC000002114285	1.39 ± 1.32	2.53 ± 1.25	2.57 ± 1.76
FDB023015	2.09 ± 1.10	2.27 ± 1.60	2.97 ± 1.39
MolPort-021-745-932	2.51 ± 1.10	1.48 ± 1.37	3.22 ± 1.31
ZINC000002159944	2.12 ± 0.88	1.09 ± 0.89	1.63 ± 1.01
SN00059335	1.25 ± 0.90	1.20 ± 0.90	2.23 ± 1.01
ZINC000002155511	2.00 ± 1.0	0.59 ± 0.78	2.46 ± 1.22
ZINC000002108239	2.72 ± 0.95	0.08 ± 0.32	2.68 ± 1.10
ZINC000002102314	2.48 ± 1.36	1.41 ± 1.03	0.46 ± 0.77
ZINC000002151580	1.52 ± 0.79	2.19 ± 2.00	0.78 ± 0.93
ZINC000002108298	0.78 ± 0.93	0.99 ± 0.96	2.23 ± 1.01
MolPort-027-852-870	1.27 ± 0.92	0.72 ± 0.67	1.44 ± 1.14
MolPort-002-515-240	1.57 ± 1.57	0.88 ± 0.99	0.13 ± 0.58
MolPort-027-852-900	0.77 ± 0.8	0.55 ± 0.80	0.10 ± 0.46
SN00236224	0.79 ± 0.85	0.29 ± 0.75	1.52 ± 1.19
SN00341524	2.51± 1.08	0.01 ± 0.80	1.75 ± 1.28
ZINC000072325799	2.78 ± 1.20	0.43 ± 0.94	0.26 ± 0.71
ZINC000095559555	0.68 ± 0.92	0.80 ± 0.97	0.35 ± 0.66
ZINC000096296967	0.77 ± 0.86	0.18 ± 0.51	1.44 ± 0.96

*Hydrogen bond counts values are shown with standard deviation*.

**Table 4:** Hydrogen-bonding residues obtained by hydrogen bond-lifetime analysis.

**Compounds**	**Hydrogen-bonding residues in RBD**
ZINC000002128789	Arg403, Arg408, Gln409, Lys417, Tyr453, Tyr473, Tyr489, Gln493, Tyr495, Gly496, Gln498, Asn501, Gly502, Tyr505
ZINC000002114285	Arg346, Ser349, Arg403, Arg408, Lys417, Tyr421, Lys444, Gly446, Tyr449, Tyr453, Arg466, Thr470, Tyr473, Tyr489, Gln493, Ser494, Gly496, Gln498, Asn501, Tyr505
FDB023015	Ser375, Thr376, Lys378, Arg403, Arg408, Gln414, Lys417, Asn437, Tyr449, Tyr453, Tyr489, Gln493, Ser494, Gly496, Gln498, Thr500, Asn501, Gly502, Val503, Gly504, Tyr505, Tyr508
MolPort-021-745-932	Ser375, Thr376, Lys378, Arg403, Arg408, Gln414, Lys417, Asn437, Tyr449, Tyr453, Tyr489, Gln493, Ser494, Gly496, Gln498, Thr500, Asn501, Gly502, Val503, Gly504, Tyr505, Tyr508.
ZINC000002159944	Arg403, Gly502, Asn501, Gly496, Tyr495, Gln498, Gln493, Unk198, Tyr505, Lys417, Tyr453, Tyr449, Ser494, Arg408, Thr500
SN00059335	Arg403, Arg408, Lys417, Tyr421, Lys444, Gly446, Tyr449, Tyr453, Lys458, Tyr473, Tyr489, Gln493, Gly496, Gln498, Thr500, Asn501, Gly502, Val503, Gly504, Tyr505

**Figure 4:**
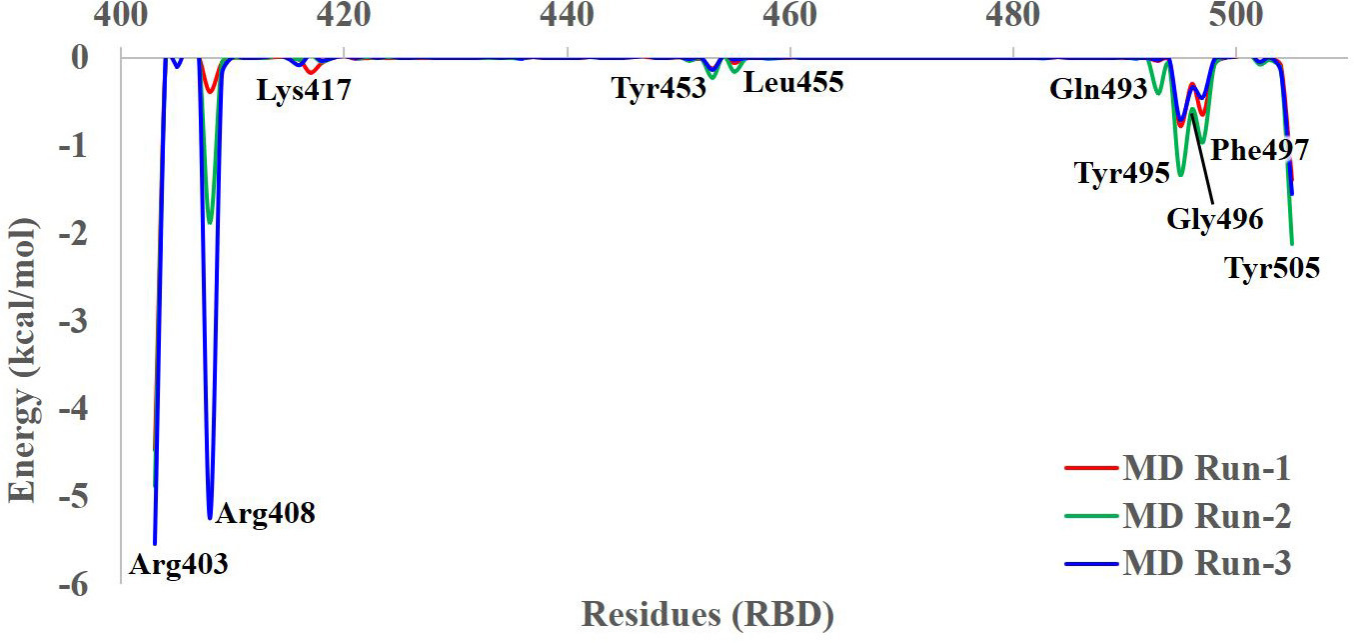
Residue-wise contributions for MM/GBSA binding free energy of ZINC000002128789 in the three MD replicates.

**Figure 5:**
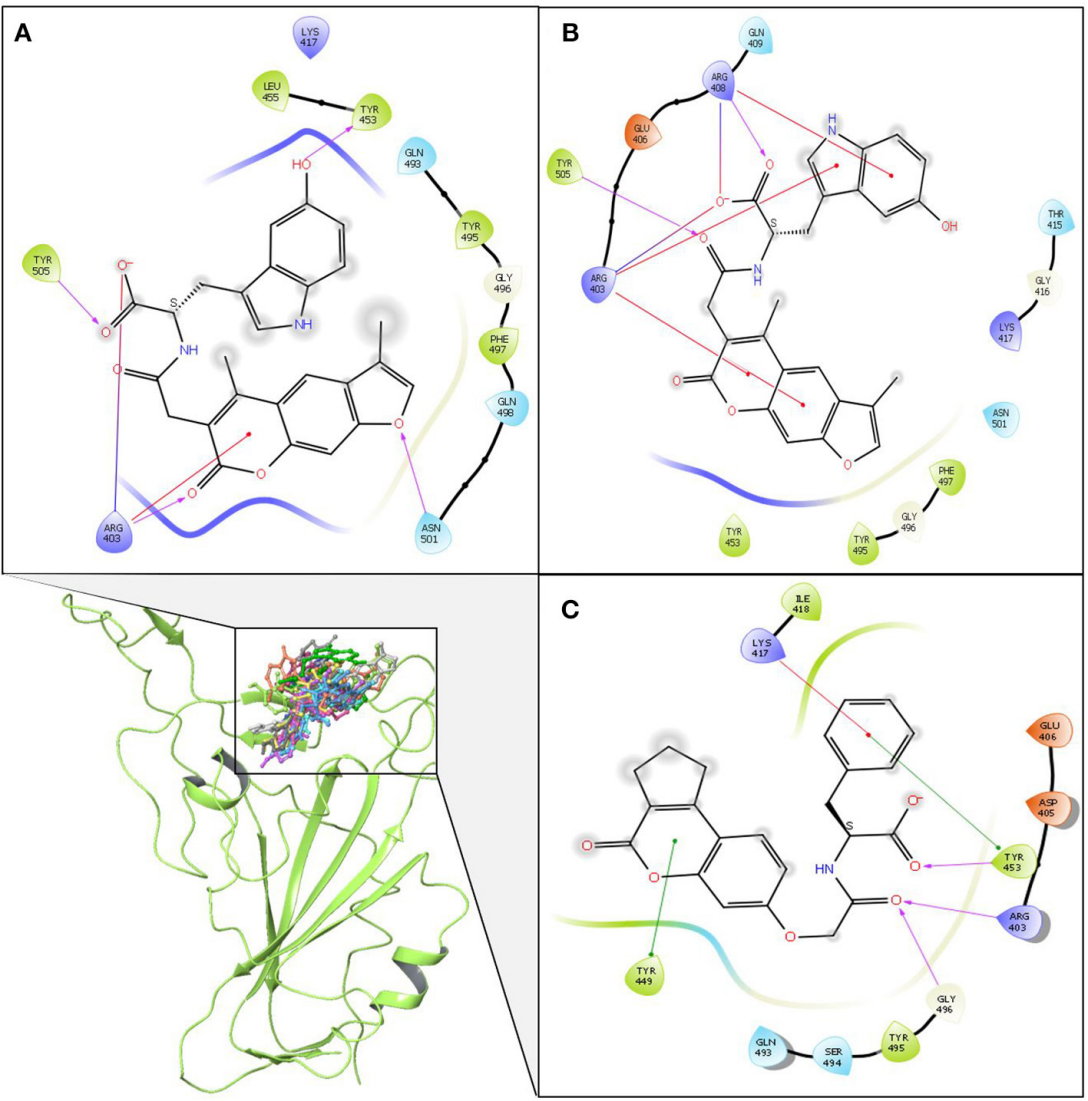
Schematic representation of detailed atom interactions of ZINC000002128789 at 100 ns of MD replicate 1 **(A)** 2 **(B)** and 3 **(C)**. Structure were obtained from all three replicates at 25 ns interval and shown as compounds cluster in ribbon model. Ligand interaction diagram is obtained for snapshot obtained from final frame. Pink, red oval and green oval arrows represent Hydrogen bond, π-Cation and π-π stacking interaction. Interacting amino acids are differentiated with various spheres. Charged, hydrophobic, polar residues are shown in blue, green and cyan while Glycine is represented with ivory spheres, respectively.

The Porcupine plot and hydrogen bond analysis of the simulations confirms that ZINC000002128789, ZINC000002159944, and SN00059335 remain stably bound in RBD interface, and they maintain contacts with ACE2–Spike protein binding interface residues. Furthermore, MM/GBSA calculations were performed with an ensemble of all three replicates to estimate the binding affinity of these compounds. The snapshots were recorded at 50 ps intervals, and these snapshots were used for ensemble-average MM/GBSA calculation to estimate the binding affinity of selected NPs. Based on MM/GBSA, the, ean ensemble-average total binding affinities (ΔG_bind_) of ZINC000002128789, ZINC000002159944 and SN00059335 are −20.82 ± 5.10 kcal/mol, −13.88 ± 6.87 kcal/mol and, −11.60 ± 10.77 kcal/mol, respectively (Supplementary Table 1). Mean ΔG_bind_ of all three replicates shows that compound ZINC000002128789 has highest binding affinity (ΔG_bind_ = −20.82 ± 5.10 kcal/mol) among three compounds and residues Arg403, Arg408, and Tyr505 largely contribute to the binding free energy of ZINC000002128789 (Figure 4). ZINC000002128789 maintains sustained contacts with Arg403, Arg408, and Tyr505 in all replicates of ZINC000002128789–RBD simulation (Figure 5). Arg403 play a role in stabilizing the interface through water mediated indirection with Asn33/His34/Glu37/Asp38 of ACE2. Arg408 and Tyr505 have been reported to interact with human ACE2 and contributes to higher affinities (Ali and Vijayan, 2020; Mittal et al., 2020).

Many natural products have shown to inhibit coronavirus with the unknown mechanism of action (Xiu et al., 2020). Few computational studies have also identified promising natural compounds to block viral entry by targeting spike protein. Wahedi et al. reported that resveratrol (ΔG_bind_ MM-GBSA= −23.88 kcal/mol) can be promising anti-COVID-19 drug candidates acting through disruption of the spike protein among other stilbenoid analogs (Piceatannol, Pinosylvin, Pterostilbene, and Chloroquine) (Wahedi et al., 2020). Chen et al. screened Thioflexibilolide A (Binding Energy: −9.2 kcal/mol) and Candidine (Binding Energy: −9.0 kcal/mol) as best compounds from 2000 natural compounds (Chen et al., 2020). Kar et al. performed docking study with natural compounds from *Clerodendrum* spp., and reported that Taraxerol (ΔG_bind_ prime MM-GBSA = −45.19 kcal/mol) as most promising inhibitory candidate against the SARS-CoV-2 spike protein (Kar et al., 2020). All the above reported compounds have shown to interact with amino acids in the ACE2-RBD interface region. In our study, selected compound ZINC000002128789 also shown stable interaction with interface residues which was confirmed by three MD simulation replicates. ZINC000002128789 and top ranked compounds from phytochemical database shares similar interactions with residues in RBD domain (Jani et al., 2020). In our study, the selected compound ZINC000002128789 maintain stable contact with Arg403, Arg408 and Tyr 505 in all three MD runs. Calculated ensemble-average ΔG_bind_ of two replicates (MD run 2 = −22.89 ± 10.93 kcal/mol; MD run 3 = −24.57 ± 22.96 kcal/mol) are higher than ΔG_bind_ of reported natural product resveratrol (Supplementary Table 1).

### ZINC000002128789 on ACE2-RBD Interface

**Figure 6:**
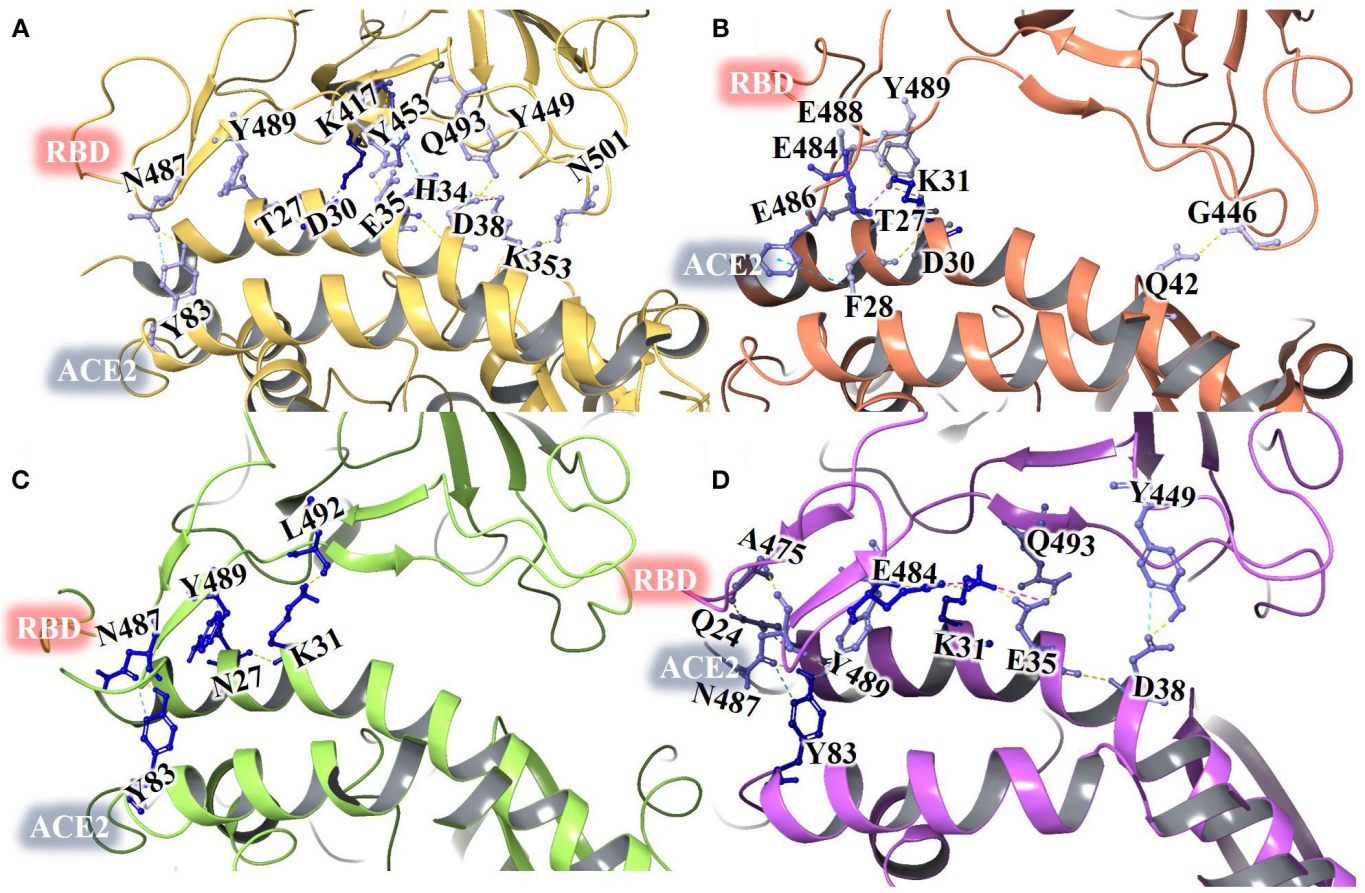
Protein interface interactions of crystal structure of ACE2-RBD of SARS-CoV-2 spike protein **(A)** and ACE2- ZINC000002128789 bound RBD structure obtained from MD run 1 **(B)** run 2 **(C)** and run 3 **(D)**.

**Figure 7:**
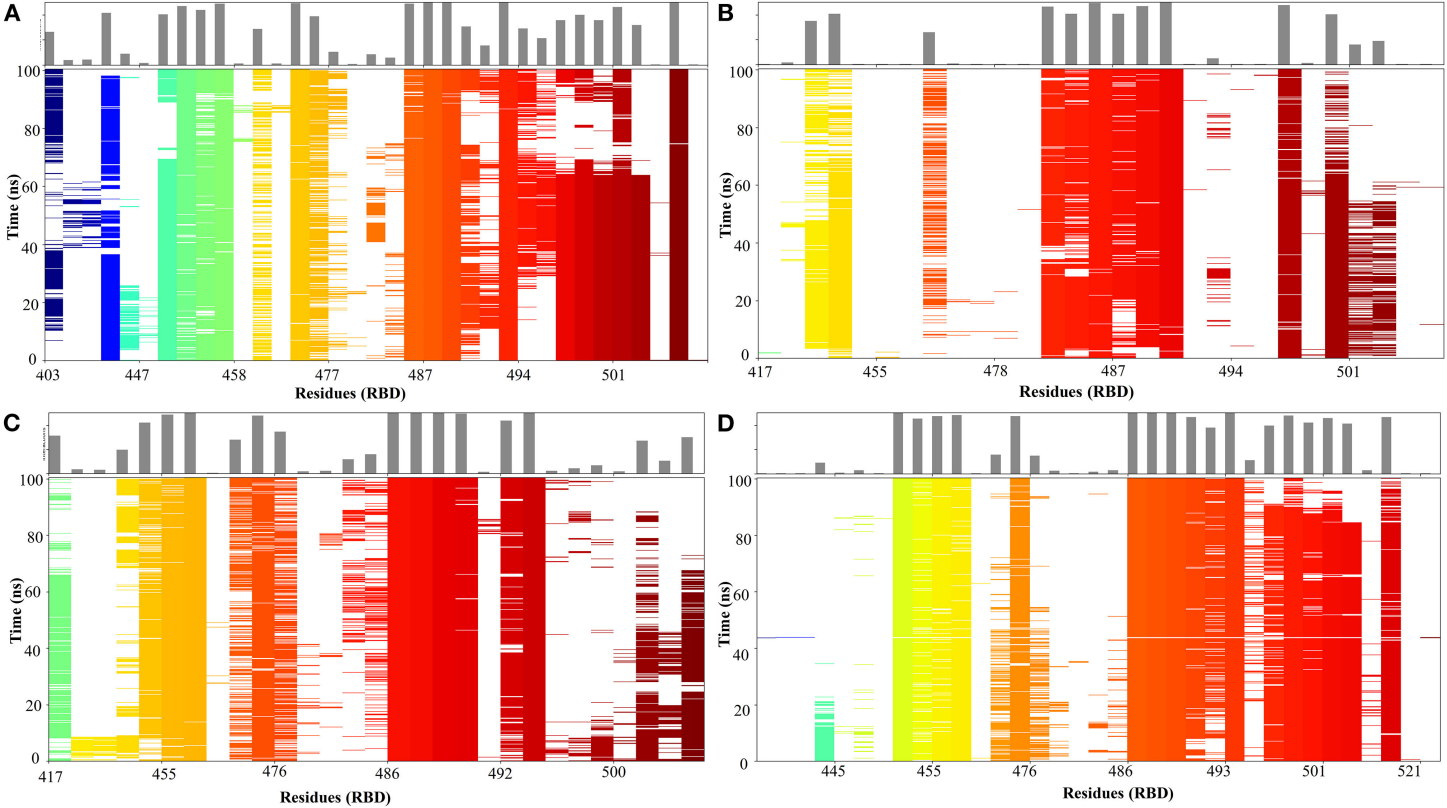
Time-dependent Protein-protein interaction matrix of crystal structure of ACE2-RBD of SARS-CoV-2 spike protein **(A)** and ACE2- ZINC000002128789 bound RBD structure obtained from MD run 1 **(B)** 2 **(C)** and 3 **(D)**. The colored plot shows the presence of interactions as a function of the time and residue number and the top plot shows interactions count for each residue in the RBD.

A 100 ns MD simulation was performed with crystal structure of ACE2-RBD and ACE2- ZINC000002128789 bound RBD. Three different ZINC000002128789 conformers were obtained from the final snapshot of MD replicates, and the crystal structure (PDB: 6M0J) coordinates were used to get similar RBD bound ACE2 with docked ZINC000002128789 in interface region. A sum of 500 snapshots were obtained at 200 ps interval and structures were used to analyze the interactions at the ACE2-RBD interface region using BioLuminate. Total number of contacts in ACE2-RBD interface region is decreased in the ZINC000002128789 bound RBD complexes (Figure 6). Further, stability of contacts was checked. Interaction fingerprints were generated for any contacts that are possibly formed between interface residues, and interaction matrix was obtained to examine the sustained interaction between ACE2–RBD interface regions (Figure 7). Ali and Vijayan reported that Lys417, Tyr449, Phe456, Tyr473, Ala475, Phe486, Tyr489, Gln493, and Gln498 interact with ACE2 through stable hydrogen bonds, hydrophobic interactions and salt bridges (Ali and Vijayan, 2020). Herein, Tyr453, Phe456, Ala475, Phe486, Asn487, Tyr489, Gln493, Asn501, Tyr 505 in RBD maintains stable contacts (> 90% of simulation time) with ACE2. While ZINC000002128789 bound complex shows only two (Phe486, Tyr489) stable contacts with ACE2 in three MD runs. A large number of stable contacts could be associated with a higher binding affinity of SARS-CoV-2 (Ali and Vijayan, 2020). ZINC000002128789 has stable interactions with Arg403, Arg408, and Tyr505, and contact matrix shows that these residues have not maintained contacts with ACE2 residues. This suggests that ZINC000002128789 blocks the major contacts between ACE2 and RBD, and thus, might be able to interfere with ACE2-RBD complex formation.

Overall, Compounds ZINC000002128789, ZINC000002159944, and SN00059335 are stable in RBD interface and ZINC000002128789 top ranked with the highest binding affinity prediction. These compounds maintain stable contacts with residues that mediate the entry of SARS-CoV-2 through RBD-ACE2 interface. Accordingly, these compounds could be potent S-protein—ACE2 interaction modulators, and based on our results, the effect of at least these three compounds should be experimentally tested.

## Conclusion

Over the years, NPs have shown a remarkable effect in the treatment of SARS-CoV-1, MERS-Cov, HIV, Influenza, Dengue and other viruses. NPs are considered as a safe and effective source in the treatment of SARS-CoV-2 and its related symptoms. Receptor binding domain in the S-protein mediates the fusion of SARS-CoV-2 with the cellular membrane through RBD-ACE2 interface. S-protein is a potential target for preventing SARS-CoV-2 entry into the human cell. In our study, we screened NPs targeting S-protein to block SARS-CoV-2 entry.

We used a protocol that combines MixMD simulation with HTVS. Selected compounds from PANTHER/ShaEP based NIB rescoring were subjected to classical MD simulations to verify the stability and affinity of binding. This protocol suggests that ZINC000002128789, ZINC000002159944, and SN00059335 would bind to RBD, and especially, ZINC000002128789 is predicted to be a potent NP to hinder the entry of SARS-CoV-2 by blocking the S-protein–ACE2 interaction.

## Data Availability Statement

The raw data supporting the conclusions of this article will be made available by the authors, without undue reservation.

## Author Contributions

OP, KG, and EJ designed the study. KG performed all calculations, except MixMD simulations and their analysis, which were performed by EJ. Preparation of NP-library was performed by SK. All authors contributed to the manuscript and approved the submitted version.

## Conflict of Interest

The authors declare that the research was conducted in the absence of any commercial or financial relationships that could be construed as a potential conflict of interest.
