# Supplementary material for: Screening of Natural Products Targeting SARS-CoV-2–ACE2 Receptor Interface – A MixMD Based HTVS Pipeline
Source: Front Chem. 2020 Nov 19;8:589769. doi: 10.3389/fchem.2020.589769 (PMC7717977; doi:10.3389/fchem.2020.589769)
Supplement: Supplementary file 1 [file Table_1.DOCX]

Supplementary Material

Screening of natural products targeting SARS-CoV-2 - ACE2 receptor interface - A MixMD based HTVS pipeline

Krishnasamy Gopinath, Elmeri M. Jokinen, Sami T. Kurkinen and Olli T. Pentikäinen*

Faculty of Medicine, Integrative Physiology and Pharmacology, Institute of Biomedicine, University of Turku, FI-20520 Turku, Finland

## Supplementary Figures


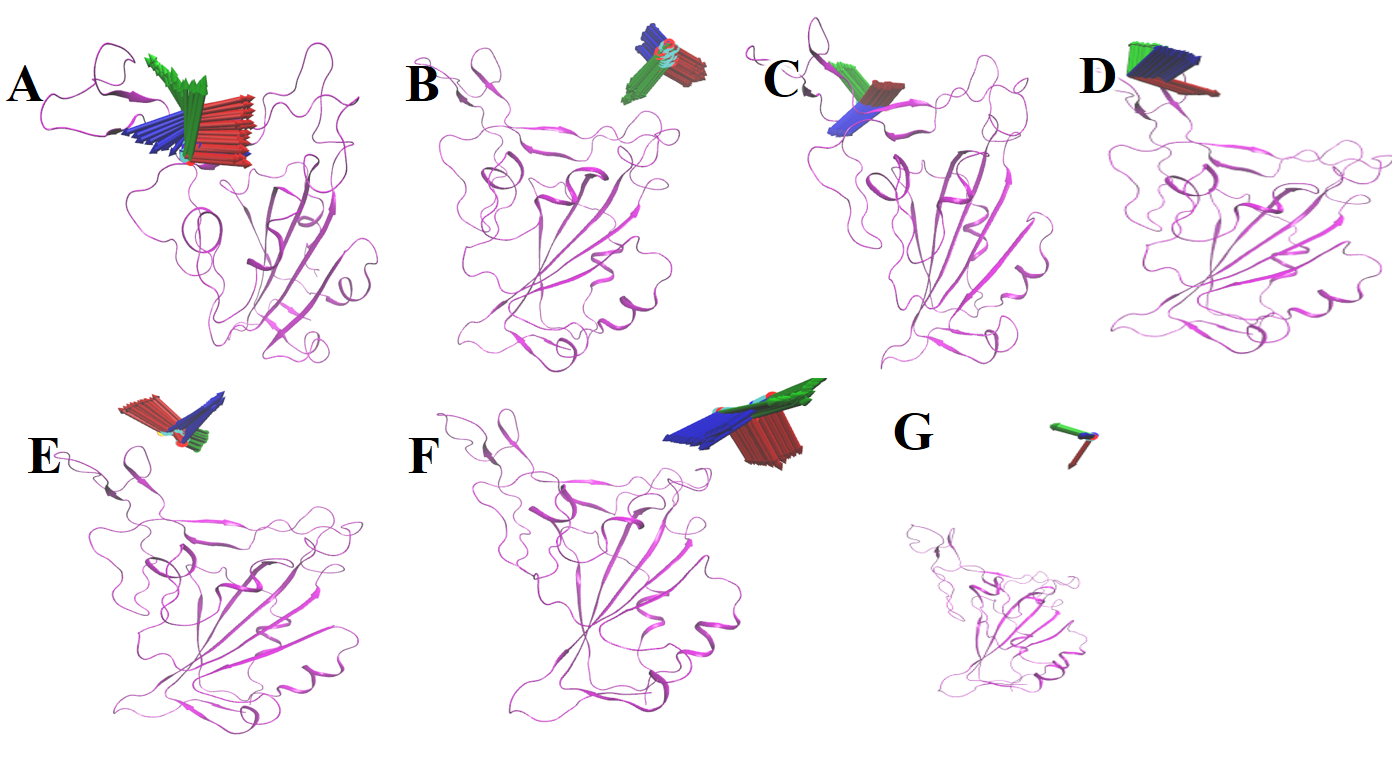


**Supplementary Figure 1.** Porcupine plots of first three eigenvectors for three MD simulation replicates of ZINC000002102314 (A); SN00236224 (B); MolPort-027-852-900 (C); ZINC000095559555 (D); SN00341524 (E); ZINC000072325799 (F); MolPort-002-515-240 (G). The arrows present on the protein complex indicate the direction and magnitude of the motion.


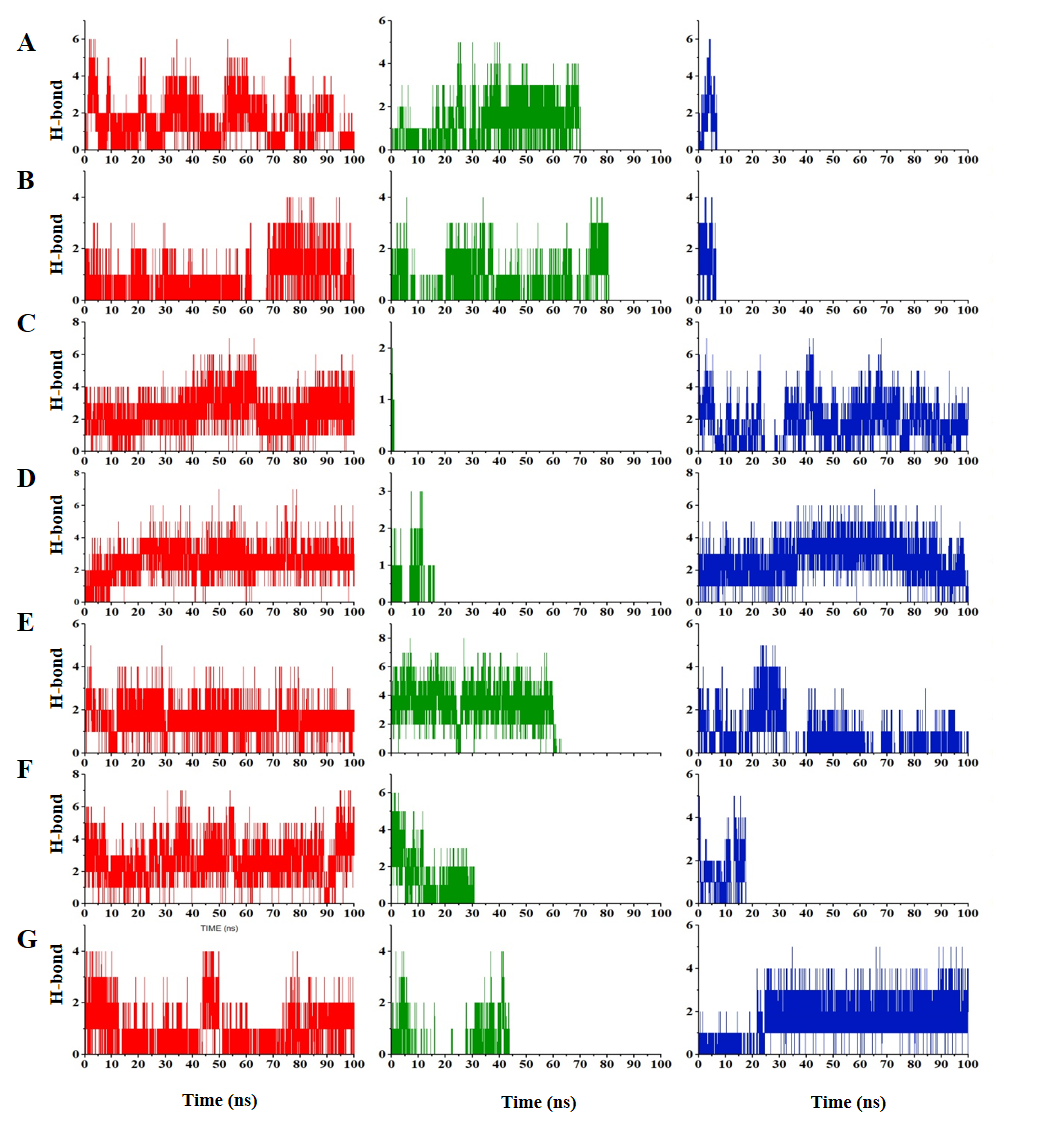


**Supplementary Figure 2.** Hydrogen bond plot. Number of hydrogen bonds formed between S-protein and MolPort-002-515-240 (A); MolPort-027-852-900 (B); SN00341524 (C); ZINC000002108239 (D); ZINC000002151580 (E); ZINC000072325799 (F) during entire simulation period , where compounds X axis shows Time in ns and Y axis shows the number of hydrogen bond formed between receptor and ligand in MD run 1 (Red) 2 (Green) and 3 (Blue).


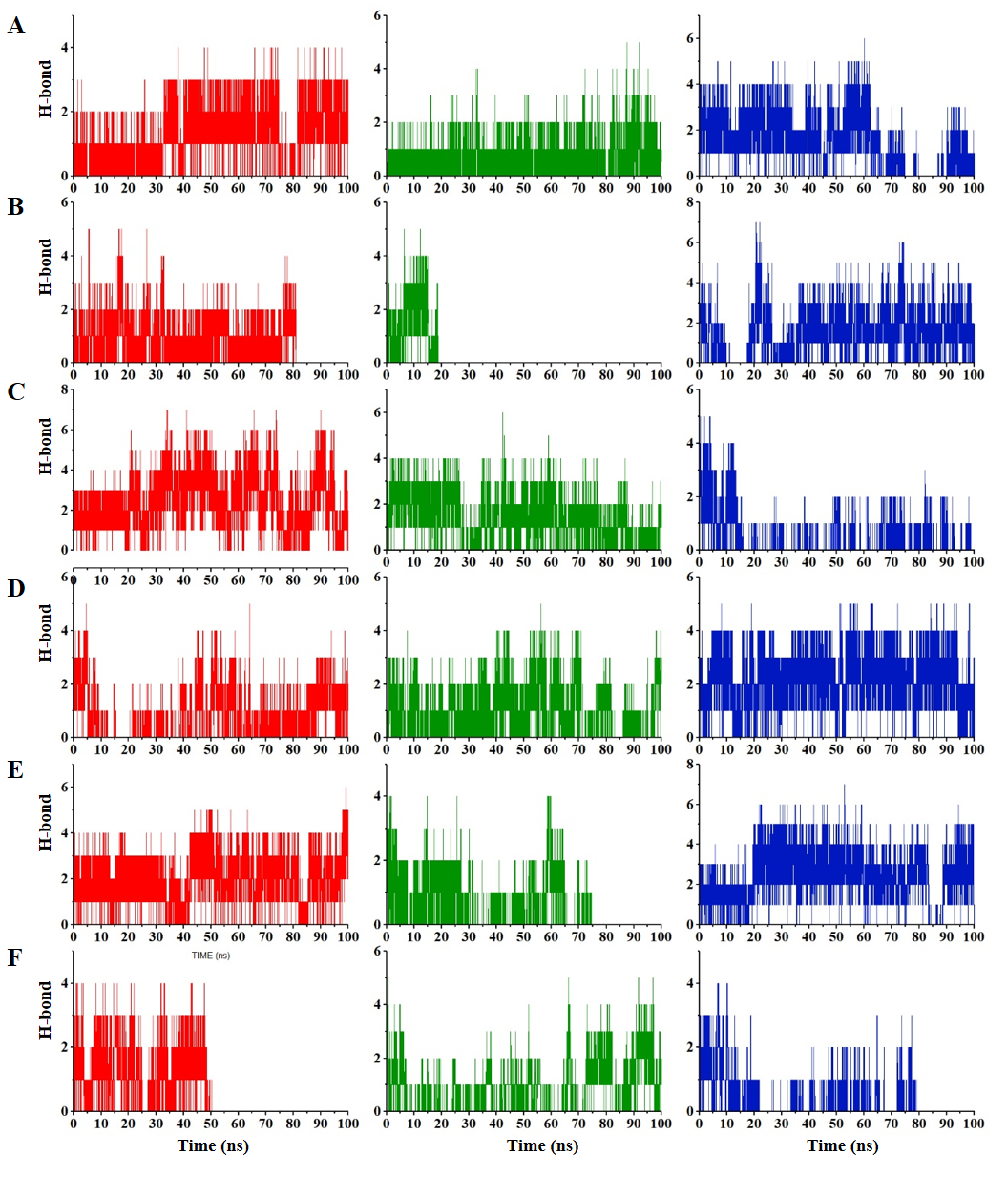


**Supplementary Figure 3.** Hydrogen bond plot. Number of hydrogen bonds formed between S-protein and MolPort-027-852-870 (A); SN00236224 (B); ZINC000002108298 (C); ZINC000002155511 (D); ZINC000095559555 (E) during entire simulation period , where compounds X axis shows Time in ns and Y axis shows the number of hydrogen bond formed between receptor and ligand in MD run 1 (Red) 2 (Green) and 3 (Blue).

## Supplementary Tables

Supplementary Table 1: Ensemble-average MM/GBSA ΔG_bind_ of stable compounds in all three MD runs.

| **Compounds** | **MD run 1^*^** | **MD run 2^*^** | **MD run 3^*^** | **Mean ΔG _bind_^*^** |
| --- | --- | --- | --- | --- |
| ZINC000002128789 | -15.00 ± 13.99 | -22.89 ± 10.93 | -24.57 ± 22.96 | -20.82 ± 5.11 |
| ZINC000002114285 | -17.85 ± 13.21 | -17.46 ± 17.29 | -18.84 ± 17.37 | -18.05 ± 0.71 |
| FDB023015 | -14.22 ± 12.75 | -13.64 ± 14.80 | -21.85 ± 13.99 | -16.57 ± 4.58 |
| MolPort-021-745-932 | -20.11 ± 14.85 | -13.26 ± 9.12 | -9.83 ± 9.73 | -14.40 ± 5.24 |
| ZINC000002159944 | -19.49 ± 13.77 | -6.22 ± 7.63 | -15.95 ± 13.12 | -13.88 ± 6.87 |
| SN00059335 | -8.54 ± 10.50 | -2.70 ± 5.67 | -23.58 ± 14.77 | -11.61 ± 10.77 |

^*^MM/GBSA ΔG_bind_ values are shown in kcal/mol with standard deviation
